# Environmental Biofilms in Livestock Production Systems: Reservoirs of Pathogens and Antimicrobial Resistance

**DOI:** 10.3390/life16060888

**Published:** 2026-05-25

**Authors:** Alexandra Ban-Cucerzan, Adriana Morar, Kálmán Imre

**Affiliations:** 1 Faculty of Veterinary Medicine, University of Life Sciences “King Mihai I” from Timisoara, 300645 Timisoara, Romania; alexandra.ban-cucerzan@usvt.ro (A.B.-C.); kalmanimre@usvt.ro (K.I.); 2 Research Institute for Biosecurity and Bioengineering, University of Life Sciences “King Mihai I” from Timisoara, 300645 Timisoara, Romania

**Keywords:** environmental biofilms, livestock production, antimicrobial resistance, biosecurity, drinking water systems, sanitation engineering, risk-based framework

## Abstract

Environmental biofilms are persistent structural components of livestock production systems and represent under-recognized drivers of pathogen persistence and antimicrobial resistance (AMR). This review examines the engineering, ecological, and operational factors that promote biofilm formation in dairy, poultry, and swine environments, with emphasis on drinking water distribution systems, feeding infrastructure, housing surfaces, and waste channels. Biofilms develop preferentially in low-shear zones, dead ends, and aging materials, where they enhance microbial tolerance to sanitation and facilitate horizontal gene transfer. Conventional monitoring approaches, largely based on planktonic sampling and single-time-point testing, underestimate attached biomass and fail to capture spatial heterogeneity. Although molecular and sensor-based technologies provide improved resolution, their farm-level implementation remains limited by cost, standardization challenges, and the absence of validated operational thresholds. Current EU surveillance frameworks focus primarily on antimicrobial use and resistance prevalence in animal isolates, while environmental compartments are rarely incorporated as monitored system elements. This review proposes a proportionate, risk-based approach that integrates existing farm data streams such as antimicrobial use metrics and biosecurity scoring systems with targeted environmental assessment of high-risk infrastructure. Mitigation strategies emphasize mechanical disruption, combined chemical sanitation, hydraulic optimization, material selection, and infrastructure lifecycle management. Embedding environmental biofilm control within existing engineering and stewardship frameworks supports more resilient, systems-based management of infectious and AMR risks in livestock production.

## 1. Introduction

Environmental biofilms are surface-attached microbial communities embedded in a self-produced extracellular polymeric substance (EPS) matrix composed primarily of polysaccharides, proteins, lipids, and extracellular DNA. This matrix confers structural integrity and functional heterogeneity, enabling microorganisms to persist under fluctuating physicochemical conditions and repeated stress exposure [1,2,3,4].

The three-dimensional EPS “matrixome” anchors cells to abiotic surfaces and regulates diffusion, cell–cell signaling, and local microenvironments, thereby enhancing tolerance to disinfectants, antimicrobials, and environmental stressors [3,4,5]. As a result, biofilm-associated cells frequently exhibit markedly greater survival capacity than their planktonic counterparts, even in systems subject to routine sanitation.

Biofilms represent the dominant microbial lifestyle in most natural and engineered moist environments, where they are highly adapted to oligotrophic conditions, variable hydrodynamics, and repeated disturbance [1,6]. In applied settings such as food production, healthcare, and agriculture, surface properties, nutrient availability, organic loading, temperature, and biofilm age jointly promote adhesion and maturation on abiotic materials, while progressively reducing susceptibility to cleaning and disinfection [4,7,8,9]. Continuous moisture availability, repeated organic inputs, complex infrastructure, and sustained microbial introduction create interconnected niches that favor long-term biofilm persistence. Routine sanitation rarely eliminates established biofilms, allowing these communities to shape the long-term microbial ecology of production environments [10,11,12,13].

The persistence of these biofilms has important implications for animal health, antimicrobial resistance, internal biosecurity, and food safety [9,10,14,15,16,17]. Understanding how biofilms establish, persist, and interact with management practices is therefore essential for interpreting disease recurrence, antimicrobial use patterns, and the limitations of conventional control strategies in livestock systems.

This review synthesizes current evidence on environmental biofilms in livestock production systems, with a focus on: (i) the ecological and structural features that promote biofilm persistence, (ii) key biofilm-forming microorganisms relevant to animal health and hygiene, (iii) the role of biofilms in the maintenance and dissemination of antimicrobial resistance, and (iv) the consequences for biosecurity, productivity, and welfare. Particular attention is given to detection challenges and to realistic EU-aligned frameworks for risk assessment and mitigation that acknowledge biofilms as inherent components of livestock production environments.

This narrative review synthesizes foundational and recent literature on environmental biofilms in livestock production systems, with particular emphasis on dairy, poultry, and swine environments. Relevant studies were identified through targeted searches conducted in PubMed, Web of Science, Scopus, ScienceDirect, and Google Scholar using combinations of keywords and controlled vocabulary related to environmental biofilms, livestock production, antimicrobial resistance, sanitation, water systems, and farm biosecurity. Search terms included combinations of “biofilm”, “environmental biofilm”, “livestock”, “dairy”, “poultry”, “swine”, “pig”, “cattle”, “drinking water system”, “waterline”, “milking equipment”, “feeding system”, “housing surface”, “drain”, “wastewater”, “manure”, “antimicrobial resistance”, “antimicrobial resistance genes”, “AMR”, “ARG”, “disinfection”, “sanitation”, “cleaning-in-place”, “biosecurity”, and “One Health”.

Studies were considered eligible when they addressed biofilm formation, persistence, microbial composition, sanitation tolerance, antimicrobial resistance, pathogen survival, environmental monitoring, or mitigation in livestock, food-production, water-distribution, or closely related farm and processing environments. Priority was given to studies addressing environmental persistence, infrastructure-associated biofilms, livestock-relevant microorganisms, and practical implications for monitoring, stewardship, and risk mitigation. Studies were excluded when they focused exclusively on unrelated clinical biofilms, human medical devices, marine systems, plant biofilms, or laboratory models without clear relevance to livestock production or environmental biofilm management. Additional references were identified through backward and forward citation tracking of key papers.

## 2. Livestock Production Systems as Ideal Niches for Biofilm Persistence

### 2.1. Multispecies Biofilms and Ecological Stability

Livestock production systems provide a combination of physical, biological, and operational conditions that strongly favor the establishment and long-term persistence of surface-associated biofilms. Unlike clinical or laboratory settings, these systems are characterized by continuous or recurrent moisture, repeated organic loading, complex infrastructure, and sustained microbial introduction from animals, feed, water, and personnel. Together, these features create stable surface–environment interfaces that support microbial attachment and EPS–mediated persistence across production cycles [12,18,19].

Biofilm development in livestock environments is therefore not episodic but systemic, emerging across interconnected compartments such as drinking water distribution systems, feeding equipment, housing structures, drainage channels, and production-specific installations. These niches differ in material composition, hydrodynamics, nutrient availability, and accessibility for cleaning, yet collectively form a network of reservoirs that sustain microbial populations, despite routine sanitation [20,21].

A defining feature of environmental biofilms in livestock systems is their multispecies organization. Rather than being dominated by single pathogens, these biofilms typically consist of diverse consortia of environmental and commensal microorganisms that establish the structural and functional foundation of the community. This diversity enhances matrix production, metabolic cooperation, and resilience to environmental fluctuations, contributing to long-term ecological stability under repeated disturbance [22,23,24].

Within these multispecies matrices, pathogenic bacteria, such as *Salmonella* spp. or Shiga toxin–producing *Escherichia coli* (STEC), are typically present at low relative abundance, but benefit disproportionately from the protective properties of the biofilm environment. Experimental and field studies consistently demonstrate that pathogens embedded within diverse biofilms exhibit substantially greater tolerance to disinfectants and environmental stress than when present in monoculture biofilms, facilitating long-term survival and intermittent release [15,22,25]. Table 1 provides a comparative overview of the principal niches supporting biofilm persistence in dairy, poultry, and swine production.

**Table 1:** Comparative overview of environmental niches supporting biofilm persistence in dairy, poultry, and swine production systems.

Environmental Niche	Dairy Production Systems	Poultry Production Systems	Swine Production Systems	Key Evidence/Relevance	References
Drinking water distribution systems (pipes, lines, dead ends)	PVC/PE and stainless-steel pipelines; low-flow sections and dead ends favor biofilms; regrowth after flushing and incomplete CIP in dairy water and milk lines	Narrow PVC lines with low flow, warm water, and organic matter strongly promote biofilms in poultry drinking water systems	Plastic and metal lines with mineral and organic deposits; low flow and incomplete flushing allow persistent biofilms and pathogen reservoirs	Pipe material, hydraulic dead zones, and organic load are consistent cross-species drivers of biofilm formation and rapid regrowth after sanitation	[12,26,27,28,29,30]
Drinkers (bowls, nipples)	Open troughs/bowls accumulate saliva, feed, and feces; biofilm load correlates with impaired water quality and resistant bacteria	Nipple drinkers with low turbulence; internal biofilms difficult to access; documented reservoirs of *Salmonella*/*E. coli* with regrowth after cleaning	Nipple bowls and valves subject to saliva and feed backflow, promoting rapid recolonization from line biofilms	Animal contact, backflow, and organic residues link drinker biofilms directly to gut exposure and AMR dissemination	[12,26,30,31]
Water tanks and reservoirs	Centralized storage with warm temperatures, sediment, and stagnant zones; insufficient cleaning between fills	Header/storage tanks in broiler houses; organic load and moderate temperatures enable persistent, sanitizer-tolerant biofilms	Occasional use tanks; irregular sanitation and sediment accumulation create long-term reservoirs feeding line contamination	Stagnation and sediment consistently emerge as key risk factors for biofilm stability and reseeding of distribution lines	[12,26,30,31,32]
Feeding systems and feed-contact surfaces	Feed troughs and milk-contact surfaces exposed to moisture and residues; multispecies biofilms act as major recontamination sources	Feeders accumulate dust, fine particles, and condensate; rough and porous surfaces favor pathogen survival	Liquid feeding systems rich in carbohydrates; incomplete drainage and internal roughness promote dense, persistent biofilms	Organic residues combined with intermittent wetting–drying cycles underpin chronic biofilm formation and recontamination	[22,27,28,32,33,34]
Milking/ production- specific equipment	Milking liners, hoses, gaskets, joints, pipelines, and tanks; complex geometries and cyclic wet–dry conditions; high biofilm loads even after CIP, especially at retainers and outlets	Not applicable	Not applicable	Dead ends, gaskets, valves, and non-CIP-covered surfaces are repeatedly identified as biofilm “hot spots” and persistent contamination sources	[27,28,32,33,35,36,37]
Housing floors and contact surfaces	Concrete holding areas with moisture and manure; floor drains and retainers host diverse mixed biofilms	High stocking-density floors and walls with heavy fecal load; dust-associated biofilms persist after disinfection	Concrete pen floors and partitions constantly soiled; slurry splashes and microcracks support stable mixed communities	Cracks, drains, and dust-exposed surfaces consistently harbor mixed biofilms that protect pathogens against sanitizers	[22,30,32,34]
Bedding or litter interfaces	Moist bedding–floor interfaces trap organic matter and reseed teats and equipment	High-porosity litter with nutrients and moisture maintains diverse biofilms, including pathogens	Limited bedding; wet floor–manure interfaces become primary biofilm sites	Porous, organic-rich interfaces act as long-term reservoirs interacting with water, air, and animal microbiota	[12,22,27,34]
Drainage systems and waste channels	Drains, channels, and waste tanks maintain continuous moisture and load; major biofilm hot spots	House drains and cooling-pad return water overlooked; residual biofilms survive between flocks and spread upward	Slurry channels and pits with constant nutrient input and rare cleaning support dense biofilms and AMR exchange	Across sectors, drains and waste lines show high diversity, sanitizer tolerance, and central roles in pathogen persistence	[22,32,34]
Operational hygiene limitations (cross-cutting)	CIP shadow zones, aging materials, complex geometries, limited internal inspection allow residual matrix and recolonization	Inaccessible internal pipe and drinker surfaces; partial cleaning between flocks; reliance on chemical dosing without mechanical action	Safety and access constraints; high organic load dilutes and inactivates disinfectants	Design flaws, incomplete CIP coverage, and organic load repeatedly undermine sanitizer efficacy across systems	[12,22,26,27,28,29,32,38]

Legend: CIP = cleaning-in-place; PVC = polyvinyl chloride; PE = polyethylene; AMR = antimicrobial resistance.

### 2.2. Drinking Water Distribution Systems

Drinking water distribution systems (DWDS) represent one of the most consistent and widespread niches for biofilm formation in livestock production. More than 90% of microbial biomass in DWDS is typically attached to internal pipe surfaces rather than suspended in bulk water, confirming biofilms as the dominant microbial lifestyle within these networks. The microbial communities inhabiting DWDS biofilms differ substantially from those detected in bulk water or animal fecal samples, reflecting selection pressures unique to the waterline environment [39].

Internal pipe walls, dead ends, valves, and pressure-regulating components create low-shear microenvironments that favor microbial attachment and EPS-protected growth [39,40,41].

Hydraulic regimes shape biofilm architecture rather than determining presence or absence. Low or intermittent flow promotes thicker and more porous biofilms, whereas higher shear stress results in thinner but denser layers rather than removal [39,41]. Combined with moderate temperatures and intermittent water use typical of livestock facilities, these conditions strongly support biofilm persistence within waterlines and drinkers [18,31].

Pipe material further influences biofilm biomass and composition. Plastic and polymer-based materials can leach biodegradable organics that enhance biofilm growth and enrichment of opportunistic taxa compared with more inert materials [40,42,43,44]. Accordingly, poultry studies frequently report dense biofilms dominated by *Pseudomonas*, *Stenotrophomonas*, and *Acinetobacter* on PVC-based systems despite routine disinfection [18,45].

Because internal pipe surfaces are rarely inspected or sampled, DWDS function as long-term, often undetected reservoirs that repeatedly expose animals throughout production cycles. Waterline biofilms frequently harbor opportunistic pathogens and antimicrobial resistance genes and represent ecological reservoirs distinct from animal fecal microbiota [19,31].

### 2.3. Production-Specific Equipment and Internal Surfaces

Production equipment introduces additional high-risk niches for environmental biofilms due to repeated exposure to warm temperatures, nutrient-rich residues, complex geometries, and material aging. In dairy systems, milking liners, hoses, joints, pipelines, and storage tanks are exposed to milk residues that rapidly form conditioning films on stainless steel, plastics, and rubber components, strongly enhancing microbial attachment [27,37]. Heat-denatured milk proteins further intensify fouling and microbial adhesion [27].

Despite cleaning-in-place (CIP) protocols, multispecies biofilms containing spoilage and pathogenic bacteria, including *Pseudomonas* spp., *Listeria monocytogenes*, and St*aphylococcus aureus,* are frequently detected on product-contact materials [27,46,47]. Pipelines, dead-legs, and bulk tank outlets act as persistent hotspots that contaminate milk with microorganisms and heat-stable enzymes, reducing shelf life and safety [48].

Material aging plays a central role in persistence. Rubber components and aging polymers support higher biofilm loads than stainless steel or glass due to increased surface roughness and microdamage, which limit detergent contact and mechanical removal [37,47,49].

In swine production, liquid feeding systems represent analogous environments. High organic loads, incomplete drainage, and prolonged feed residence times promote stable biofilms dominated by fermentative microorganisms, leading to feed quality deterioration. Although intensive cleaning can temporarily disrupt these communities, rapid recolonization limits long-term efficacy [50].

### 2.4. Housing Structures and Animal-Contact Surfaces

Housing environments provide extensive surface areas that sustain persistent biofilms under continuous organic contamination and frequent animal contact. Floors, walls, partitions, gratings, and hutches are repeatedly exposed to feces, urine, litter, dust, and secretions, creating conditions that favor rapid microbial colonization [12,51]. Field studies demonstrate that polymeric and metallic housing surfaces can develop dense biofilms within weeks of use [51].

Unlike enclosed systems, biofilm persistence on housing surfaces is driven primarily by repeated animal-mediated contamination and limited drying between production cycles. Opportunistic and pathogenic bacteria such as *Escherichia coli*, *Klebsiella* spp., *Pseudomonas aeruginosa*, and *Staphylococcus aureus* can survive for extended periods on housing materials, reinforcing their role as environmental reservoirs [30,52].

Routine cleaning and disinfection reduce surface contamination but rarely eliminate established biofilms, particularly on rough, vertical, or overhead surfaces where access and coverage are limited [12,53].

### 2.5. Key Biofilm-Forming Microorganisms in Livestock Production Systems

Across poultry, swine, and cattle operations, as well as downstream meat and dairy processing environments, a relatively consistent set of bacterial groups is repeatedly detected, indicating strong ecological selection rather than random contamination [11,19,54].

#### 2.5.1. Opportunistic Gram-Negative Biofilm Formers as Structural Scaffolds

*Pseudomonas* spp. are among the most frequently detected biofilm formers in livestock drinking water systems, milking equipment, housing surfaces, and food-processing drains [18,19,54]. Their high EPS production, quorum-sensing regulation, and tolerance to disinfectants allow them to act as structural scaffold organisms, stabilizing multispecies biofilms and protecting co-colonizing bacteria from sanitation stress [21,55,56].

Other Gram-negative genera, including *Acinetobacter* spp.*, Stenotrophomonas* spp., *Aeromonas* spp., *Psychrobacter* spp., and *Brochothrix* spp., are recurrently identified in water systems, drains, and cold or wet niches across livestock and processing environments [21,45,57,58].

#### 2.5.2. Gram-Positive Biofilm Formers Relevant to Animal Health and Hygiene

*Staphylococcus aureus* and coagulase-negative staphylococci are key biofilm formers on animal-contact surfaces, housing materials, and dairy equipment, particularly in poultry and dairy systems [36,59,60]. Biofilm-associated staphylococci exhibit increased tolerance to antimicrobials and disinfectants, contributing to chronic infections such as mastitis and to persistent environmental contamination between production cycles [55,61].

*Enterococcus* spp., including *E. faecalis* and *E. faecium*, are widely detected in swine and poultry environments and frequently demonstrate strong biofilm-forming capacity combined with multidrug or vancomycin resistance [62,63].

#### 2.5.3. Classical Zoonotic and Foodborne Pathogens Within Environmental Biofilms

Foodborne and zoonotic pathogens such as *Salmonella* spp., *Campylobacter jejuni*, *Escherichia coli*, *Listeria monocytogenes*, and *Klebsiella* spp. are frequently recovered from environmental biofilms at low relative abundance across livestock farms and processing facilities [11,15,24,64]. Although these organisms rarely dominate biofilm biomass, their survival within multispecies matrices enables long-term persistence and intermittent release, sustaining exposure even in the absence of active animal shedding [17,22,56].

In poultry waterlines, *Campylobacter* spp. and *Salmonella* spp. are repeatedly linked to biofilm reservoirs that survive between flocks, contributing to reinfection despite cleaning and downtime [11,19].

Similarly, in meat and dairy processing environments, background biofilm flora dominated by *Pseudomonas* spp. and psychrotrophic genera shelter pathogens such as *L. monocytogenes* and *E. coli* O157:H7, complicating eradication efforts [21,54,65,66].

In cattle, all major agents of the bovine respiratory disease complex (BRD), namely *Mannheimia haemolytica*, *Pasteurella multocida*, *Histophilus somni*, and *Mycoplasma bovis*, have demonstrated biofilm-forming capacity [13]. Biofilm formation by these organisms is increasingly hypothesized to contribute to chronic, treatment-refractory respiratory infections in feedlot systems [13,61].

Understanding which microorganisms underpin biofilm persistence provides a biological basis for risk-based monitoring, targeted sanitation, and mitigation strategies, reinforcing the integration of microbiological ecology into livestock biosecurity and antimicrobial stewardship frameworks [56,67]. Table 2 provides an overview of common materials in livestock production environments, their characteristic biofilm-forming microbial communities, and how these associations intersect with EU recommendations for hygienic design and risk mitigation.

#### 2.5.4. Non-Bacterial Components of Environmental Biofilms

Although bacterial communities represent the main focus of this review, environmental biofilms in livestock production systems should not be interpreted as exclusively bacterial structures, because fungi, yeasts, and viral particles, particularly bacteriophages, may be embedded within biofilm matrices and influence surface colonization, nutrient cycling, microbial interactions, pathogen persistence, and tolerance to cleaning and disinfection [12,68].

Yeasts and filamentous fungi can colonize moist, organic-rich farm niches, including milking equipment, pipelines, bedding interfaces, feed-contact surfaces, drains, and water-associated environments [12,36,69,70].

In dairy systems, yeasts have been reported as relevant members of milking-system surface biofilms, with *Candida parapsilosis* identified among dominant yeast taxa [36].

These organisms may contribute to extracellular matrix development and persistence because fungal and yeast biofilms produce matrices containing polysaccharides, proteins, nucleic acids, lipids, and other structural components that protect embedded cells against environmental stress, disinfectants, and antifungal agents [71,72,73].

In food, dairy, and livestock-associated environments, yeast and fungal biofilms on stainless steel, filtration membranes, drains, bedding materials, and other abiotic surfaces may act as persistent reservoirs of spoilage organisms and undesirable microorganisms, reducing sanitation efficacy and contributing to microbial carryover between production stages [12,54,69,74].

Non-bacterial biofilm members may also shape bacterial community structure through direct and indirect interactions, as mixed bacterial–yeast–fungal biofilms can form stratified structures, alter nutrient availability, modify local pH and oxygen gradients, and influence microbial metabolism [74,75,76].

Although much of the mechanistic evidence comes from food, fermentation, and processing environments, these interactions are relevant to livestock production because similar organic-rich, moist, and surface-associated niches occur in animal buildings, feed systems, bedding interfaces, drinkers, drains, and equipment surfaces [12,18,30,69,70].

Viral particles, particularly bacteriophages, may also be retained within or interact with extracellular polymeric substances in biofilm matrices [68,77,78].

While viruses do not form biofilms independently in the same way as bacteria, yeasts, or filamentous fungi, the biofilm matrix can influence viral retention, diffusion, persistence, and interactions with microbial hosts [68,77].

Bacteriophages may affect bacterial population dynamics and, under certain conditions, contribute to gene transfer processes, while phage-based approaches are increasingly explored as tools for disrupting polymicrobial biofilms in food, water, and livestock-related environments [54,77,78,79].

Overall, yeasts, filamentous fungi, and viral particles should be recognized as part of the broader ecology of environmental biofilms in livestock systems, although available livestock-specific evidence remains more limited than for bacterial biofilms.

**Table 2:** Livestock-system materials, associated biofilm-forming microorganisms, and implications for EU design and hygiene principles.

Material/Component	Typical Use in Livestock Systems	Biofilm-Related Risk Profile (Microorganism-Specific)	Relevant EU Design and Hygiene Principles	Key Gap Highlighted by Biofilm Evidence	References
PVC/PE plastics	Drinking water lines, feeders, flexible hoses	Support dense multispecies biofilms dominated by Proteobacteria (*Pseudomonas* spp., *Acinetobacter* spp., Enterobacteriaceae, *Legionella*, *Mycobacterium*), with frequent presence of *Enterococcus* and *Staphylococcus*; higher biomass and diversity than metals, especially in aged pipes	Materials must be non-toxic, cleanable, and allow effective sanitation	Internal aging, leaching, and roughening of plastics are not explicitly addressed	[45,51,80]
Stainless steel (AISI 304/316)	Milking equipment, pipelines, tanks, drinkers	Rapidly colonized after conditioning by *Staphylococcus* spp. (incl. *S. aureus*), *Enterococcus faecalis*, *Bacillus* spp., *Pseudomonas* spp., *Acinetobacter* spp., *Klebsiella* spp., *E. coli*; *Proteobacteria* spp. often act as structural background flora	Preferred smooth, corrosion-resistant material	Biofilm risk after organic conditioning is underestimated	[37,48,81]
Rubber, elastomers (Buna-N, EPDM, silicone)	Seals, gaskets, liners, hoses	Strongly favor *Staphylococcus aureus* biofilm formation; nitrile rubber shows significantly higher biofilm loads than steel or plastic; rapid surface degradation enhances persistence	Components must be cleanable and replaceable	Replacement intervals rarely consider biofilm risk	[47,82]
Concrete (unfinished/rough)	Floors, walls, pens, drainage channels	Highly porous surfaces support thick biofilms dominated by Firmicutes, Bacteroidetes, Proteobacteria, with documented *Campylobacter* spp., *Enterococcus* spp., *E. coli*, *Salmonella* spp. persistence	Surfaces should be hygienic and cleanable	No enforceable standards for sealing or surface finishing	[12,51]
Epoxy-coated/sealed concrete	Floors, drains, wet zones	Reduced adhesion when intact; cracks and wear allow recolonization by mixed environmental and intestinal bacteria	Recommended for wet areas	Long-term integrity not routinely verified	[21,83]
Metal drains and wastewater interfaces	Slurry channels, drains, waste zones	Persistent multispecies biofilms dominated by *Pseudomonas* spp., *Psychrobacter* sp., *Acinetobacter* spp., *Brochothrix* spp., sheltering *Salmonella* spp. and *Listeria* spp.	Cleaning and disinfection required	Drain interiors rarely included in verification	[54,57,58]
Liquid feed system materials	Swine liquid feeding pipelines	Nutrient-rich residues support stable biofilms of *Enterococcus faecalis/faecium* and *E. coli*, often MDR or VRE	No material-specific EU guidance	High-risk systems largely unmanaged	[62,63]
Bedding-contact materials	Mats, floor interfaces	Moisture retention promotes mixed environmental–fecal biofilms, including *Enterococcus*, *E. coli*, and anaerobes	Bedding hygiene emphasized	Material–biofilm interactions overlooked	[12,84]
Water tanks/reservoirs	Poultry and cattle systems	Sediment-associated biofilms dominated by Proteobacteria with frequent AMR genes; act as upstream inoculum sources	Regular cleaning recommended	Internal biofilms not routinely monitored	[12,31,85]

Legend: AISI = American Iron and Steel Institute; AMR = antimicrobial resistance; EPDM = ethylene propylene diene monomer; EU = European Union; MDR = multidrug-resistant; VRE = vancomycin-resistant enterococci.

## 3. Environmental Selection and Maintenance of Antimicrobial Resistance

In livestock production systems, environmental biofilms may contribute to AMR persistence by maintaining resistant bacteria and antimicrobial resistance genes under chronic, low-intensity selection pressures embedded in routine environmental conditions, in addition to episodic therapeutic antimicrobial use [56,67,86]. Consequently, environmental biofilms function as long-term stabilizing reservoirs of resistance, sustaining AMR across production cycles even in the absence of continuous antimicrobial use.

Environmental and drinking water biofilms in agricultural settings are consistently identified as reservoirs of antimicrobial resistance genes (ARG) and multidrug-resistant bacteria, reflecting their capacity for prolonged retention, protection from washout, and repeated exposure to sublethal stress [12,19,87,88].

Livestock production environments are routinely exposed to sub-inhibitory concentrations of antibiotics and disinfectants, arising from antimicrobial administration, residual disinfectants in water systems, manure and wastewater contamination, and incomplete cleaning and disinfection procedures [56,86,89]. Unlike therapeutic exposure, these pressures are spatially heterogeneous and temporally prolonged, generating selection gradients within environmental biofilms [67,90].

Under these conditions, antimicrobial pressure does not eliminate biofilm-associated populations but instead selectively removes susceptible cells, allowing tolerant and resistant phenotypes to persist and dominate over time [56,86]. Repeated exposure to low concentrations of biocides has been shown to induce adaptive responses such as efflux pump activation and membrane modification, frequently accompanied by cross-resistance to clinically relevant antimicrobials [87,91]. Evidence from agricultural water and waste-associated biofilms further demonstrates that ARG accumulation can occur under low but persistent antimicrobial pressure, emphasizing exposure duration rather than peak concentration as a key determinant of resistance maintenance [56,86].

The physical and physiological architecture of environmental biofilms plays a central role in stabilizing AMR once established. The EPS matrix restricts antimicrobial penetration and creates microenvironments characterized by reduced exposure intensity, oxygen limitation, and metabolic heterogeneity [10,90]. As a result, biofilm-associated cells can exhibit higher tolerance than planktonic cells without necessarily acquiring new resistance determinants [9,92].

This structural protection favors the retention and concentration of existing resistance traits, including ARG-carrying lineages and persister subpopulations, while repeated sanitation cycles preferentially remove more exposed, susceptible cells [11,12,89]. High cell density, prolonged residence time, and abundant extracellular DNA further enhance horizontal gene transfer and plasmid stability, reinforcing AMR persistence within biofilm communities over extended periods [86,90,93].

### Influence of Antimicrobial Administration Routes on Biofilm-Mediated AMR Maintenance

Antimicrobial administration routes in livestock production systems strongly shape where and how environmental selection pressure is exerted, thereby influencing the role of biofilms in AMR maintenance (Table 3). Group treatments delivered via drinking water or liquid feed introduce antimicrobials directly into infrastructure components that are intrinsically prone to biofilm formation. Consequently, antimicrobial administration via these routes exposes not only animals but also resident biofilm communities within the infrastructure to direct antimicrobial inputs [19,94,95].

In swine production, administration of sulfonamides and trimethoprim through liquid feeding systems selected for resistant *Enterobacteriaceae* within pipeline biofilms, with resistance levels remaining elevated after treatment cessation, indicating sustained contamination originating from biofilm reservoirs [94,95,96].

Importantly, antimicrobial exposure is not confined to the treatment period. Antimicrobial residues and resistant microorganisms sorb into biofilms and accumulate in manure, wastewater, and runoff, extending selection pressure beyond clinical decision-making and into connected environmental compartments, including wildlife reservoirs that may contribute to the environmental circulation of resistant bacteria [56,86,97,98,99]. As a result, antimicrobial use patterns determine not only immediate therapeutic outcomes but also the duration and spatial reach of environmental selection pressure.

**Figure 1:**
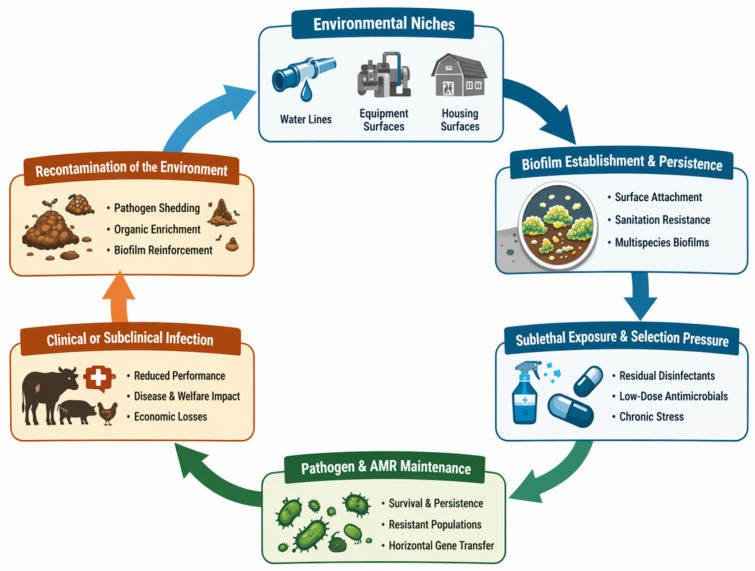
Schematic representation of contamination and reinfection cycles driven by environmental biofilms in livestock production systems.

As illustrated in Figure 1, environmental biofilms represent a critical link between antimicrobial use and reinfection cycles. Addressing this link requires shifting from short-term microbial reduction strategies toward sustained environmental management approaches that limit biofilm persistence and unintended selection pressure.

## 4. Consequences for Animal Health, Productivity, Welfare, and Biosecurity

Persistent environmental biofilms in intensive livestock systems exert cumulative effects on animal health, production efficiency, and internal biosecurity [12,17,18].

Biofilm-associated infections in livestock are difficult to eradicate, contributing to persistent mastitis, respiratory, skin, wound, and reproductive disorders across production systems [59,60]. These infections, often subclinical or low-grade, reduce growth performance, milk yield, meat quality, and reproductive efficiency, while increasing veterinary costs, culling rates, and overall production losses [59,60,85]. Beyond economic impacts, prolonged biofilm-mediated disease and repeated treatments compromise animal welfare through sustained discomfort and cumulative stress, particularly in high-density environments with continuous exposure [11,60].

### Why Control Strategies Fail in Livestock Production Systems

A primary cause of control failure is the physical and operational structure of livestock systems. Even well-designed cleaning-in-place systems cannot deliver uniform shear forces, contact times, or disinfectant concentrations across complex geometries. As infrastructure ages, surface roughness, microdamage, and material fatigue further reduce sanitation efficacy, allowing protected biofilm cores to persist despite repeated interventions [12,21,82,83].

Operational realities compound these structural constraints. Time pressure, labor availability, production schedules, and safety considerations inevitably introduce variability in cleaning execution. Small deviations in concentration, temperature, or exposure time, often unavoidable in practice, disproportionately affect biofilm control, even when protocols are nominally followed. Standardized hygiene procedures applied across heterogeneous facilities therefore produce inconsistent outcomes, masking site-specific risks rather than resolving them [11,82,83].

These surface reservoirs also create internal biosecurity vulnerabilities that facilitate pathogen dissemination within and between production units. Movement of personnel, shared equipment, contaminated water, and incomplete sanitation facilitate within-farm and between-unit dissemination when environmental reservoirs persist [100,101].

Poor microclimate control, inadequate water quality, and ineffective cleaning practices are consistently associated with higher disease incidence and increased antimicrobial use, highlighting biofilms as critical weak points in internal biosecurity [11,102].

Environmental biofilms also compromise internal biosecurity by enabling pathogen carryover between production cycles, even in systems operating under all-in/all-out management, where sanitation is assumed to reset infection risk [11,101]. This is particularly relevant in broiler production, where biofilm-associated bacteria may survive cleaning and disinfection between batches and contribute to house recolonization after chick placement [11,18,30,83].

Moreover, chronic low-dose exposure from environmental biofilms promotes subclinical carriage, allowing pathogens to circulate silently within herds and flocks while escaping routine clinical detection and biosecurity audits [11]. As a result, apparent compliance with biosecurity protocols may coexist with ongoing internal dissemination, underscoring that effective biosecurity depends not only on procedural adherence but on controlling environmental persistence within production systems [102].

To translate the qualitative impacts of environmental biofilm persistence into operationally relevant outcomes, Table 4 summarizes key system-level consequences for animal health, productivity, biosecurity, and antimicrobial stewardship, together with quantitative indicators routinely monitored within livestock production systems.

## 5. Detection and Monitoring Challenges in Farm Environments

Despite increasing recognition of environmental biofilms as critical reservoirs of pathogens and antimicrobial resistance in livestock systems, their routine detection on farms remains limited and inconsistent [51,96]. This gap reflects three interacting challenges: structural inaccessibility, methodological bias toward planktonic cells, and temporal variability.

Livestock environments contain numerous biofilm-prone surfaces, including concrete, metals, and polymer-based water and feed lines that are difficult to sample non-destructively. Conventional swabbing or plating methods recover only a fraction of attached biomass and disrupt spatial organization, yielding unrepresentative estimates of contamination [51]. No currently available detachment method reliably removes all surface-associated cells, further constraining accurate quantification. In practice, routine hygiene monitoring remains largely plankton-biased: swabs, ATP assays, and culture-based methods primarily assess detached or loosely associated cells rather than intact biofilms [51,96]. Reviews of EPS confirm that no universally validated biofilm-specific marker exists to clearly distinguish sessile from planktonic populations, and EPS analytical methods remain insufficiently standardized [107,108].

Quantitative evidence underscores the scale of under-recovery. In dry-surface biofilms, optimized foam swabs recovered approximately 30% of contamination, while cotton or viscose swabs recovered as little as 3–6% [109]. Sonication-assisted methods demonstrate substantially higher detachment efficiency compared with standard swabbing [110,111]. Microscopy further reveals that industrial biofilms form heterogeneous, clustered structures with protected “hotspots,” making friction-based sampling highly dependent on sampling location and operator technique [112]. As a result, limited or superficially selected sampling points can leave dense multispecies communities undetected, creating a misleading perception of hygiene control.

Temporal variability adds another layer of complexity. Longitudinal studies across aquatic and farm-linked systems show that biofilm biomass and composition can shift markedly over days to months in response to hydraulic changes, temperature, disturbance, and operational dynamics [113,114,115,116]. Transient release events often coincide with hydraulic demand or disturbance, meaning that single-time-point sampling frequently misses episodic shedding and generates false-negative or overly reassuring results [114,116,117].

To overcome these limitations, in situ coupon-based capture systems have been developed and successfully applied in livestock environments [51,118]. Removable standardized surfaces allow biofilms to form under realistic conditions and preserve three-dimensional structure for imaging, culture, and molecular analysis. Compared with classical swabbing, coupon-based approaches yield more reproducible and structurally representative assessments of biomass and diversity and enable longitudinal tracking of biofilm dynamics and intervention effects [12,51].

Advanced molecular and real-time analytical tools provide further resolution but remain largely research-bound. High-throughput sequencing, metagenomics, and resistome analyses are challenged in farm settings by low microbial biomass, host DNA interference, and complex organic matrices that complicate extraction and interpretation [35,119,120]. Methodological variation in sample processing can substantially alter detected signals, limiting comparability across studies [121]. Although resistome profiling offers conceptual advantages for linking biofilms to AMR risk, harmonized surveillance pipelines remain limited [11,60].

Mass spectrometry-based approaches also provide valuable high-resolution tools for biofilm characterization, although their use in routine livestock farm monitoring remains limited. LC-MS/MS can support detailed profiling of extracellular polymeric substances, including proteins, lipids, polysaccharide-associated compounds, and metabolites involved in biofilm maturation, stress tolerance, and antimicrobial or biocide exposure responses [4,122]. MALDI-TOF MS has been widely used for rapid microbial identification and has additional value for comparing biofilm-associated microbial phenotypes, while MALDI imaging mass spectrometry can spatially map metabolites, lipids, signaling molecules, and antimicrobial-associated compounds within structured microbial communities [122,123]. These platforms are particularly relevant for linking biofilm structure with functional traits such as EPS composition, lipidomic adaptation, metabolomic activity, and AMR-associated biochemical signatures [4,123]. However, their translation to farm-level surveillance is constrained by cost, sample preparation complexity, matrix effects from manure, feed residues, bedding, and water sediments, and the lack of standardized workflows or operational thresholds for interpreting biofilm-associated risk in livestock environments [122].

Similarly, emerging in situ technologies, including electrochemical impedance spectroscopy, optical interferometry, and microsensors, demonstrate high sensitivity for continuous, non-destructive biofilm detection under controlled conditions [117,124,125,126,127,128]. However, field-scale validation in livestock facilities remains rare, and few systems define operational thresholds linking signal changes to actionable management decisions [5,129]. Consequently, a translational gap persists between advanced biofilm analytics and farm-level decision-making.

This monitoring gap extends to policy frameworks. Current EU and national AMR surveillance systems emphasize antimicrobial use, resistance in clinical or animal isolates, and health outcomes, while environmental biofilms are rarely incorporated as monitored compartments [96,130]. Environmental research consistently identifies biofilms as hotspots of horizontal gene transfer, yet surveillance designs typically focus on bulk water or clinical isolates rather than attached communities [56,67,97,98,131].

Evidence from wastewater and aquatic systems demonstrates that sub-inhibitory antibiotic residues and co-selective agents can sustain ARG selection within biofilms even after reductions in antibiotic inputs [56,86,132]. Biofilm matrices stabilize resistomes over time, buffering short-term fluctuations and serving as long-term reservoirs capable of reseeding surrounding communities. Within this ecological framework, stable or rising resistance despite reduced antimicrobial use is consistent with delayed ecological response and ongoing low-level selection within persistent biofilm reservoirs. Without explicit integration of environmental biofilms into One Health surveillance architectures, distinguishing true temporal lag from active biofilm-driven AMR maintenance remains difficult [19,67,86,97].

### Practical EU-Aligned Framework for Environmental Biofilm Risk Assessment

Effective management of environmental biofilms in livestock production systems requires monitoring approaches that are feasible at the farm level, proportionate to operational constraints, and compatible with existing EU benchmarking, biosecurity, and antimicrobial stewardship frameworks [11,133]. This approach is consistent with Regulation (EU) 2019/6, which updated EU rules on veterinary medicinal products and supports responsible veterinary antimicrobial use, and with EU-level recommendations emphasizing improved animal health, hygiene, biosecurity, husbandry, and preventive management as core measures for reducing the need for antimicrobial treatment [102,134,135,136]. Rather than relying on exhaustive testing, which is rarely sustainable or informative in complex farm settings, a risk-based framework that integrates routinely collected farm indicators with targeted assessment can provide actionable insights while minimizing disruption to production [11,12].

EU livestock systems already generate extensive data streams through antimicrobial use (AMU) monitoring, AMR surveillance, productivity benchmarking, and biosecurity scoring tools, which together form a robust foundation for early risk detection [133,135,136,137].

The European Sales and Use of Antimicrobials for Veterinary Medicine (ESUAvet) annual surveillance reports provide harmonized data from EU and European Economic Area countries and support monitoring of progress toward prudent antimicrobial use in animals [138]. Similarly, the JIACRA IV report integrates antimicrobial consumption and resistance data from humans and food-producing animals across the EU/EEA, illustrating how AMU and AMR trends can be interpreted jointly under a One Health framework [139]. The EU Summary Report on antimicrobial resistance in zoonotic and indicator bacteria further provides harmonized AMR monitoring data from humans, food-producing animals, and food, including *Salmonella* spp., *Campylobacter jejuni*, and *Campylobacter coli* from relevant livestock sectors and derived meat products [140].

Risk-based biosecurity scoring systems, such as Biocheck.UGent quantify, internal and external biosecurity and allow benchmarking across farms, while integrated platforms such as ClassyFarm combine AMU, AMR, animal welfare, and biosecurity indicators to classify farms by risk and guide interventions [133,141,142]. Poultry biosecurity databases across Europe further demonstrate how standardized checklists can feed national surveillance and benchmarking, while also highlighting heterogeneity in implementation and gaps in data use [143].

Within this context, the first tier of the proposed framework relies on routine benchmarking indicators already embedded in EU surveillance and stewardship programs, including treatment incidence and retreatment frequency, the proportion of group antimicrobial treatments, key productivity metrics such as somatic cell count, average daily gain, and feed conversion efficiency, and available AMR trend data from routine diagnostics [133,141,142]. Monitoring these indicators longitudinally allows farms and advisors to detect deviations from baseline performance that may signal unresolved environmental infection pressure rather than isolated clinical events [11,102]. Threshold-based interpretation, such as sustained increases in treatment incidence, persistent failure to reduce AMU despite stewardship efforts, or deterioration of productivity across consecutive monitoring periods, provides a transparent and reproducible trigger for further investigation [133,141].

When Tier 1 indicators suggest elevated risk, the framework escalates to a second tier of targeted environmental assessment designed to confirm or refute suspected biofilm persistence [12,35]. Rather than broad environmental sampling, this tier focuses on predefined sentinel sites known to support stable biofilms, such as drinking water systems, high-risk equipment interfaces, and persistently wet housing or drainage surfaces [11,12]. This approach reflects growing evidence that only a limited subset of structural sites act as long-term biofilm hotspots, while most surfaces contribute little to sustained risk [12,35]. Interpretation emphasizes patterns over absolute counts, with recurrent detection after cleaning, inconsistent results relative to sanitation history, and fluctuating signals indicative of biofilm-mediated release rather than transient contamination [11,12].

A core element of the framework is structured intervention verification, recognizing that apparent short-term improvements may mask rapid biofilm regrowth [11,12]. Sentinel sites are assessed before interventions, shortly after implementation, and again after a defined regrowth period to determine whether reductions are sustained or transient, thereby distinguishing effective mitigation from cosmetic sanitation effects [11]. Persistent rebound across monitoring points indicates the need for infrastructural, design, or management-level changes rather than further intensification of cleaning or antimicrobial use [12,144].

Environmental findings are interpreted alongside AMU, AMR, and animal health indicators to support integrated stewardship and biosecurity decisions [133,141]. Evidence from cattle and broiler systems shows that improved internal biosecurity is associated with lower antimicrobial use, although effective solutions are highly farm-specific and require coordinated action across hygiene, infrastructure, and management [102,133,141,145]. Within this framework, persistent environmental signals combined with stable or increasing AMR trends prioritize environmental and infrastructural mitigation, whereas concurrent improvement in environmental indicators and reduced treatment incidence supports continuation of current strategies [11,142].

Annual review and system optimization close the monitoring loop by identifying recurring hotspots, updating sentinel sites and thresholds, and prioritizing long-term structural or maintenance interventions as system characteristics evolve [133,142,143]. By framing environmental biofilms as operational risk factors rather than isolated hygiene failures, this framework embeds biofilm management within routine benchmarking and stewardship workflows already accepted across EU livestock systems [11,12].

Overall, this two-tier, EU-aligned framework translates the complex ecology of environmental biofilms into a practical decision-support tool that links routine farm indicators with targeted verification and structured response [133,142]. By focusing on trends, thresholds, and intervention verification rather than exhaustive testing, it provides a realistic pathway for integrating environmental biofilm risk into antimicrobial stewardship, biosecurity assessment, and long-term farm management planning.

**Figure 2:**
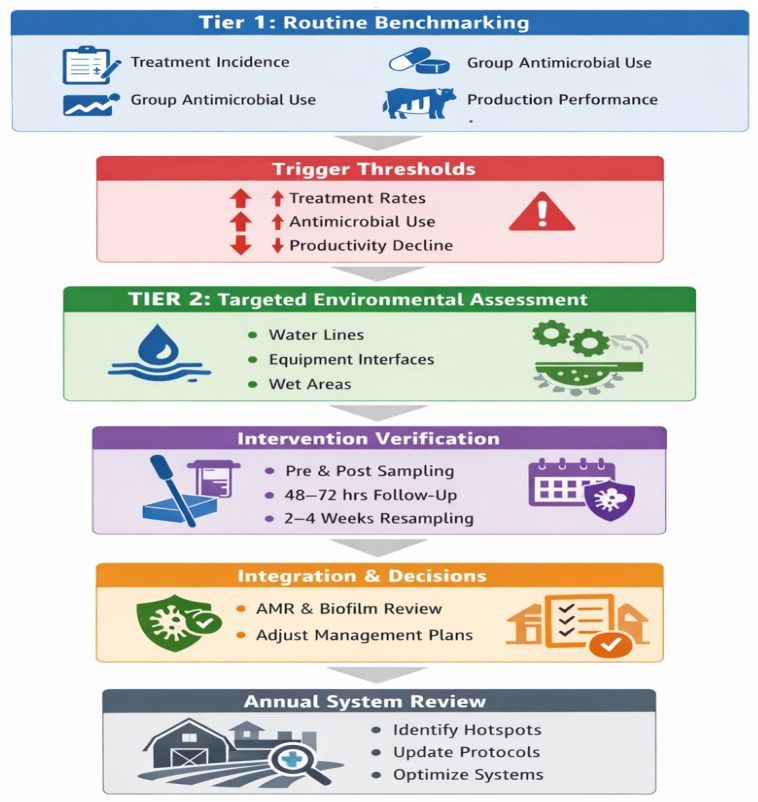
EU-aligned framework for environmental biofilm risk assessment in livestock systems.

Figure 2 summarizes the tiered, EU-aligned monitoring framework and illustrates how routine performance indicators, targeted environmental assessment, and stewardship decisions are linked within a practical biofilm risk management cycle.

## 6. Mitigation Approaches Within Realistic Farm Constraints

Because environmental biofilms are structurally embedded in livestock production systems, complete eradication is rarely achievable under real farm conditions, making risk reduction rather than elimination the practical objective [11,12,14]. Effective mitigation, therefore, requires risk-based strategies that reduce biofilm persistence, limit AMR maintenance, and disrupt reinfection cycles while remaining compatible with economic, labor, and infrastructure constraints [102].

Infrastructure design strongly influences long-term biofilm risk, and even in existing facilities, targeted design-oriented adjustments can meaningfully reduce persistence [12,21]. Reducing dead ends in waterlines, improving drainage to prevent stagnation, and prioritizing smoother, wear-resistant materials limit the formation of protected biofilm niches without requiring full system redesign [21,95,129]. Equipment renewal strategies should account for hygiene degradation over time, as aging seals, hoses, and connectors frequently become stable biofilm reservoirs [11,12]. Regular flushing, consistent disinfectant dosing, and verification of residual concentrations can reduce biofilm-associated microbial release even when complete removal is not achieved [95,146].

## 7. Knowledge Gaps and Future Perspectives

Biofilm persistence reflects a disconnect between engineering and microbiological research, with infrastructure design, fluid dynamics, material aging, and microbial ecology often studied in isolation. Evidence increasingly indicates that effective mitigation requires integrated approaches that jointly address design constraints, maintenance strategies, and microbial behavior [13,35,51,96,147]. Bridging this gap will be critical for developing interventions that are both technically robust and practically deployable on farms.

Another persistent gap is the scarcity of longitudinal, system-level studies. Most available research remains cross-sectional, providing snapshots of biofilm presence or resistance profiles rather than capturing persistence, regrowth, and response to interventions across production cycles [13,35,51,96]. Longitudinal studies that follow environmental biofilms through sanitation events, management changes, and infrastructure aging are needed to distinguish transient contamination from stable reservoirs and to evaluate mitigation strategies under realistic operational variability.

Looking forward, environmental biofilm management should be framed as a continuous risk-management process rather than a problem with a definitive solution. Future progress will depend on integrating environmental biofilm risk into routine benchmarking, strengthening verification and feedback mechanisms, and adopting adaptive management strategies that reflect system-specific constraints. Aligning research, monitoring, and farm-level decision-making with the operational realities of livestock production offers the most realistic pathway to reducing the long-term impact of environmental biofilms on animal health, AMR, and food safety.

## 8. Conclusions

From a One Health perspective, environmental biofilms represent critical interfaces linking animal health, environmental management, and food safety, contributing to recurrent exposure, antimicrobial reliance, and potential downstream transmission. Their management is therefore essential not only for farm-level performance but also for broader public health and environmental objectives.

Within the European Union, existing stewardship and surveillance frameworks provide strong tools for monitoring antimicrobial use, resistance, and productivity, yet their explanatory power remains limited without explicit consideration of environmental persistence. Integrating biofilm-related risk into benchmarking, intervention verification, and system-level decision-making would strengthen these frameworks without requiring fundamental structural change.

Overall, effective control requires a shift from eradication-focused approaches toward realistic, system-level biofilm management based on targeted monitoring, infrastructure-aware mitigation, and continuous verification. Recognizing biofilm reservoirs as predictable features of livestock systems is central to achieving sustainable improvements in animal health, antimicrobial stewardship, and long-term production resilience.

## Figures and Tables

**Table 3:** Environmental biofilms as AMR selection and maintenance niches in livestock production systems in relation to antimicrobial use patterns.

Environmental Niche	Main Exposure Route/AMR Selection Pressure	Typical AMR-Related Mechanism in Biofilms	Main Consequence for Livestock Systems	References
Drinking water distribution systems (pipes, lines, dead ends)	Group antimicrobial treatments via drinking water; residual disinfectants; low-flow conditions promoting prolonged contact	Direct exposure of established pipe biofilms to sub-inhibitory antimicrobial and biocide concentrations; enrichment of tolerant and resistant populations; persistence of ARG in attached biomass	Continuous reseeding of drinking water with resistant bacteria; prolonged selection pressure even after treatment cessation; repeated animal exposure	[19,56,94,95]
Drinkers (bowls, nipples)	Backflow of saliva, feed particles, fecal contamination, and medicated water residues	Recolonization from upstream line biofilms combined with local retention of resistant bacteria in protected wet niches	Direct oral exposure of animals to resistant microorganisms; local amplification of resistant populations at animal–water contact points	[12,30,31,95]
Water tanks and reservoirs	Irregular cleaning; sediment accumulation; retention of antimicrobial residues and disinfectants	Sediment-associated biofilms act as upstream reservoirs where resistant bacteria and ARG persist and reseed distribution lines	Repeated downstream contamination of water systems; difficulty eliminating resistant populations through routine flushing alone	[12,31,87]
Feeding systems and feed-contact surfaces	Antimicrobials delivered through liquid feed; nutrient-rich residues; intermittent wetting and incomplete drainage	Stable biofilms exposed to prolonged antimicrobial contact select for resistant Enterobacteriaceae and enterococci; biofilms protect survivors after treatment	Sustained contamination of feeding infrastructure; repeated exposure of animals to resistant bacteria; persistence after therapy	[22,56,86]
Liquid feeding pipelines (swine-specific)	Direct administration of antimicrobials via liquid feed	Selection of resistant Enterobacteriaceae within pipeline biofilms; persistence of resistance after the end of treatment	Infrastructure-mediated AMR maintenance linked directly to management practice	[93,94,95]
Milking/production-specific equipment	Repeated sanitation with possible sublethal biocide exposure; residual organic matter; incomplete CIP coverage	Biofilms on internal surfaces protect bacteria from disinfectants and may co-select for biocide tolerance and cross-resistance	Persistent contamination of product-contact surfaces; difficulty removing resistant environmental flora from dairy systems	[27,37,89]
Housing floors and contact surfaces	Repeated contamination with manure, dust, wastewater, and disinfectant residues	Mixed multispecies biofilms retain resistant bacteria and support survival under fluctuating sanitation pressure	Environmental persistence of MDR bacteria on animal-contact surfaces; recurrent exposure between cleaning cycles	[11,12,87]
Bedding or litter interfaces	Chronic organic loading; fecal deposition; indirect exposure to antimicrobials excreted by treated animals	Moist organic interfaces support mixed biofilms that protect resistant fecal bacteria and facilitate persistence outside the host	Silent environmental maintenance of resistant bacteria near animals; possible recolonization route	[12,56,86]
Drainage systems and waste channels	Manure, wastewater, runoff, antimicrobial residues, and disinfectant carryover	High-density biofilms in drains and slurry channels favor ARG retention, horizontal gene transfer, and survival under chronic low-dose exposure	Major environmental reservoir for AMR maintenance and redistribution within and beyond the farm	[56,86,96,97]
Operational hygiene limitations (cross-cutting)	Incomplete cleaning, inaccessible surfaces, high organic load, suboptimal biocide dosing	Repeated sublethal exposure removes susceptible cells while protected biofilm-associated populations persist; promotes adaptation and possible cross-resistance	Failure of sanitation to reduce AMR burden; long-term stabilization of resistant populations in infrastructure	[11,38,89,91]

Legend: AMR = antimicrobial resistance; ARG = antimicrobial resistance genes; MDR = multidrug-resistant; CIP = cleaning-in-place.

**Table 4:** Operational indicators reflecting the system-level consequences of environmental biofilm persistence in livestock production systems.

Indicator Category	Operational Indicator	Relevance to Environmental Biofilm Persistence	Applied Interpretation	References
Antimicrobial use	Treatment incidence rate (treatments per animal or per cycle)	Persistent biofilms sustain recurrent infection pressure, increasing treatment frequency	Elevated treatment incidence may indicate unresolved environmental reservoirs rather than isolated prescribing decisions	[11,17,102]
	Retreatment/relapse frequency	Reinfection from biofilm reservoirs necessitates repeated antimicrobial courses	High retreatment rates suggest failure to eliminate persistent sources of contamination or infection	[55,60,61]
	Proportion of group treatments	Background exposure from environmental biofilms may favor repeated mass medication	High reliance on group treatment may reflect persistent exposure not adequately controlled by hygiene and infrastructure management	[19,94]
Animal health and productivity	Somatic cell count (dairy systems)	Continuous low-level exposure from biofilms contributes to subclinical mastitis	Elevated or unstable SCC values may indicate persistent environmental contamination affecting udder health	[12,59,60]
	Average daily gain	Chronic biofilm-mediated exposure reduces growth efficiency	Reduced ADG may reflect sustained infection pressure rather than acute, short-term disease events	[60,84,85]
	Feed conversion efficiency	Subclinical disease and chronic inflammation impair nutrient utilization	Declining feed efficiency may signal underlying environmental health constraints linked to persistent biofilm reservoirs	[11,102]
Biosecurity and hygiene	Recurrent positive environmental samples	Biofilms survive sanitation and recolonize surfaces and water systems	Repeated positive findings suggest persistence rather than sporadic hygiene failure	[12,21,24]
	Persistence of identical isolates across cycles	Environmental biofilms act as stable microbial reservoirs	Recovery of similar isolates over time supports the presence of internal contamination sources not eliminated between cycles	[15,103,104]
	Increased sanitation frequency without improvement	Biofilm resilience limits the effectiveness of repeated cleaning	Lack of improvement despite intensified sanitation suggests a mismatch between hygiene effort and infrastructure design or biofilm location	[11,53,105]
Antimicrobial resistance	Stable or increasing AMR despite reduced use	Biofilms maintain resistant populations independently of current antimicrobial input	AMR trends may lag behind stewardship efforts because resistant populations persist within environmental reservoirs	[9,56,67]
	Discrepancy between clinical success and AMR trends	Short-term treatment success may coexist with long-term resistance persistence	Highlights the need to integrate environmental monitoring into AMR assessment and interpretation	[93,106]
System-level efficiency	Increased downtime between production cycles	Extended sanitation may be required to compensate for persistent biofilms	Longer downtime can reflect hidden operational costs associated with persistent environmental contamination	[12,17,21,24]
	Performance variability within cohorts	Uneven spatial exposure to environmental reservoirs may produce heterogeneous outcomes	Marked within-cohort variability may suggest localized biofilm hotspots within the production environment	[21,24]

Legend: ADG = average daily gain; SCC = somatic cell count; AMR = antimicrobial resistance. The table summarizes operational indicators that may reflect unresolved environmental biofilm persistence within livestock production systems and supports their interpretation in the context of biosecurity, antimicrobial stewardship, and production performance.

## Data Availability

No new data were created or analyzed in this study.

## Abbreviations

The following abbreviations are used in this manuscript:

ADG	Average daily gain
AISI	American Iron and Steel Institute
AMR	Antimicrobial resistance
AMU	Antimicrobial use
ARG	Antimicrobial resistance genes
ATP	Adenosine triphosphate
BRD	Bovine respiratory disease
CIP	Cleaning-in-place
DNA	Deoxyribonucleic acid
DWDS	Drinking water distribution systems
EEA	European Economic Area
EPDM	Ethylene propylene diene monomer
EPS	Extracellular polymeric substances
ESUAvet	European Sales and Use of Antimicrobials for Veterinary Medicine
EU	European Union
JIACRA	Joint Inter-Agency Antimicrobial Consumption and Resistance Analysis
LC-MS/MS	Liquid chromatography–tandem mass spectrometry
MALDI-TOF MS	Matrix-assisted laser desorption/ionization time-of-flight mass spectrometry
MDR	Multidrug-resistant
MS	Mass spectrometry
PE	Polyethylene
PVC	Polyvinyl chloride
SCC	Somatic cell count
STEC	Shiga toxin-producing *Escherichia coli*
VRE	Vancomycin-resistant enterococci
